# Robust Pan/Tilt Compensation for Foreground–Background Segmentation

**DOI:** 10.3390/s19122668

**Published:** 2019-06-13

**Authors:** Gianni Allebosch, David Van Hamme, Peter Veelaert, Wilfried Philips

**Affiliations:** 1 TELIN-IPI, Ghent University, Sint-Pietersnieuwstraat 41, B-9000 Gent, Belgium; david.vanhamme@ugent.be (D.V.H.); peter.veelaert@ugent.be (P.V.); wilfried.philips@ugent.be (W.P.); 2 imec, Kapeldreef 75, B-3001 Leuven, Belgium

**Keywords:** PTZ camera, camera parameters, motion compensation, foreground–background segmentation

## Abstract

In this paper, we describe a robust method for compensating the panning and tilting motion of a camera, applied to foreground–background segmentation. First, the necessary internal camera parameters are determined through feature-point extraction and tracking. From these parameters, two motion models for points in the image plane are established. The first model assumes a fixed tilt angle, whereas the second model allows simultaneous pan and tilt. At runtime, these models are used to compensate for the motion of the camera in the background model. We will show that these methods provide a robust compensation mechanism and improve the foreground masks of an otherwise state-of-the-art unsupervised foreground–background segmentation method. The resulting algorithm is always able to obtain $F_{1}$ scores above 80% on every daytime video in our test set when a minimal number of only eight feature matches are used to determine the background compensation, whereas the standard approaches need significantly more feature matches to produce similar results.

## 1. Introduction

Pan-tilt-zoom (PTZ) cameras provide maximum coverage of a scene with a single camera. They expand the level of flexibility, since the operator can select the desired camera viewpoint at runtime, which is not possible with standard fixed-camera solutions. PTZ cameras are therefore applied in numerous applications, such as the detection of people in prohibited areas or in the counting of vehicles.

To further automate these applications, the interesting objects (foreground) first need to be separated from less interesting ones (background). This process is called *foreground–background segmentation*. Many foreground–background (FGBG) segmentation algorithms have been proposed in the literature and an extensive overview will be presented in Section 2. However, coping with a panning or tilting camera still poses a challenge for these algorithms, and thus the potential of automated PTZ camera analysis currently remains unfulfilled.

Most FGBG segmentation methods are built on the following principle: a model of the appearance of the background is learned and constantly updated. Given this model, a region in the image is classified as ‘foreground’ in locations where the current appearance differs significantly from the modeled appearance. As long as the camera position and orientation remain static, the background model can be locally tuned (whether automatically or not) to handle certain challenging conditions, such as dynamic backgrounds or changing illumination. However, once the camera starts rotating or tilting, it becomes difficult to adjust the entire background model to continue detecting foreground objects accurately, and standard motion compensation techniques are not sufficiently robust to overcome this issue [1,2].

The specific, restricted nature of the motion of PTZ cameras offers some interesting solutions to this problem. In this paper, we show that the apparent motion of points on the image plane can be described in a rigorous mathematical framework. We will demonstrate that by analyzing feature correspondences for a given panning camera regarding this framework, a more robust motion compensation method and thus more reliable foreground masks can be constructed. The three main contributions of this paper can be summarized as follows:

1. We propose a novel method which automatically determines the parameters that are necessary to model a panning camera (i.e., the initial tilt angle and focal length) at runtime. No specific calibration objects (e.g., markers or checkerboard patterns) need to be used. Only feature tracks corresponding to background objects need to be extracted.
2. We demonstrate two novel camera motion compensation methods, capable of coping with purely panning or pan/tilt motions of cameras, respectively. These methods estimate the motion between two successive frames from several matches, by exploiting the calculated camera parameters mentioned above. We will demonstrate that these compensation mechanisms are very robust, even when the camera motion is estimated from a low number of feature matches.
3. The panning camera model and compensation frameworks are embedded into the state-of-the-art FGBG segmentation algorithm described in [3], using it to compensate for the pan/tilt motion in the background model when necessary. Compared to the original (affine-based) compensation mechanism and a full 8 degree of freedom homography-based compensation, the proposed mechanism delivers notably higher $F_{1}$ scores for a low number of feature matches.

In the next section, we present an overview of relevant methods related to foreground–background segmentation and camera motion compensation. In Section 3, a description of the camera model and an overview of the notations used are presented. Then, in Section 4 mathematical models for a purely panning camera and a simultaneously panning and tilting camera respectively are derived. The proposed panning camera framework allows us to determine the focal length and (fixed) tilt angle, which is demonstrated in Section 5. Once these parameters are known, the proposed model can be used further to compensate for the motion of the camera between successive frames, which is treated in Section 6. The integration in the FGBG segmentation method is described in Section 7. Experiments and the evaluation of the algorithm are treated in Section 8.

## 2. Related Work

Recently, there has been a surge in high-performing semi-supervised video segmentation methods. These methods typically combine spatiotemporal features and semantic information (e.g., embedded in a Convolutional Neural Network or a similar deep learning architecture). The segmentation masks are inherently steered towards more probable foreground objects, such as cars, cyclists or pedestrians, using networks trained on large databases such as ImageNet [4] as a backbone. This allows them to achieve very accurate segmentation results in nearly any scenario, given that at least one manually annotated frame is available [5,6,7]. Modern unsupervised video segmentation methods are also able to accurately detect such objects by making additional assumptions about object motion or the camera setup [8], but still typically require the entire video to be processed before the output is generated.

The segmentation methods described above are suitable for applications such as automatic video annotation or fine-grained sports analysis. However, for other domains such as automatic surveillance or obstacle detection in traffic, there is a need for immediate, real-time detection of anything that can be considered noteworthy, without the possibility to first manually annotate a frame or process an entire video. An alternative and well-studied foreground-detection approach is background subtraction, where input frames are compared to a background model that is built over previous samples and maintained during the entire sequence. This approach is essentially agnostic towards which specific foreground object is present in the scene, which means any significant change in the scene can be detected in real time, regardless of object class. With the aforementioned surveillance and traffic applications in mind, the foreground–background segmentation algorithm presented in this paper is based on the background subtraction principle.

Many background subtraction-based foreground–background segmentation algorithms can be considered to be extensions to the seminal Mixture of Gaussians (MoG) method by Stauffer and Grimson [9]. The main strategy is to model the background appearance (often color or intensity) as a mixture of Gaussian distributions per pixel. Static background regions can be modeled with a small number of significant, narrow Gaussian components, while more dynamic regions are modeled by a larger number of and/or wider Gaussian distributions. Comprehensive overviews of MoG-based methods can be found in [10,11].

More recent FGBG segmentation methods diverge from the MoG-based model in favor of a more efficient representation. The ‘codebook model’ replaces the Gaussians with a collection of codes, each consisting of the typical color values, and a maximal lightness and hue divergence [12]. This enables a shorter processing time, while still providing a decent flexibility. Another category of algorithms stores the model by a collection of samples, rather than by its parameters [13,14]. This category of algorithms can model background distributions which cannot be represented accurately by a Mixture of Gaussians. Furthermore, alternative features such as image gradients [15], Local Binary Patterns [16] or derived versions of them [17,18,19] provide more robustness when the illumination changes in the scene.

The most recent improvements came in the form of adaptive background maintenance. The basic idea is to update the model more rapidly and to raise the detection threshold automatically in dynamic regions. These regions can be identified by a typically larger deviation between the input and the background model [20] or from the detection of ‘blinky’ foreground pixels [17,18]. When the camera moves however (e.g., PTZ), the background model cannot be updated in a straightforward manner. In this scenario, pixels in the input image no longer correspond to their counterparts stored at the same locations in the background model. Hence, the background model cannot be directly updated from the locally corresponding input pixels.

Image registration between the input and the modeled background provides a solution to this problem [21]. Currently, the best performing unsupervised method in the literature on the ChangeDetection.NET 2014 dataset [22], on the full category of PTZ cameras is C-EFIC [3]. To explain why classical algorithms cannot be used on PTZ cameras, we have a closer look at the adjustment mechanism it uses to specifically compensate for camera motion: C-EFIC assumes that the bulk of the scene remains static throughout the sequence. Hence, most optical flow vectors (calculated between successive frames) represent the motion of the camera. This property is exploited in two ways. First, camera motion is detected whenever significant flow vectors are found in a large portion of the current input image. Second, the optical flow vectors are used to determine the best affine transformation between images in a RANSAC framework. Specifically, C-EFIC combines a static and a dynamic camera background model. When camera motion is detected, the background images in the dynamic model are affinely transformed such that they correctly overlap the current input image, based on feature matches and RANSAC. Hence, the foreground mask is extracted from local comparison between the input and the transformed background model and this model is also updated accordingly. When no camera motion is detected, the (non-compensated) static model is used to detect foreground regions.

Though the C-EFIC method is efficient and the results are good compared to other state-of-the-art methods [23], the affine approximation only holds for points close to the principal ray [24]. This means that points near the borders of the image will not be transformed accurately, especially when wide angle lenses are used. For PTZ cameras, it can be shown that the transformation between two PTZ frames is well approximated by a homography. This assumption also remains valid in many practical situations when the distance to the scene is large in comparison with the translations of the camera [25]. Hence, foreground–background segmentation can be improved by incorporating such a transformation to compensate for camera motion [26]. The seminal method to calculate a homography transformation uses the Direct Linear Transformation (DLT) [24]. The basic DLT method computes a homography estimation by directly solving a set of eight equations, obtained from four image point correspondences. When significantly more point correspondences are available, a much more robust estimate is found in a nonlinear optimization framework.

However, such correspondences are not always available in typical applications requiring FGBG segmentation, e.g., when the background consists of large flat or dynamic regions. Yi et al. [1] and Kim and Kwon [2] argue that the ‘standard’ approach of estimating the (full 8 degree of freedom) homography from a set of feature matches as a standalone compensation mechanism is not sufficiently robust, i.e., such a compensation mechanism is not accurate enough to enable correct pixel-level comparisons between an input image and a background model in the context of foreground–background segmentation.

In these situations, reducing the number of degrees of freedom when possible provides a potential solution. Li et al. [27] exploit the specific nature of pan/tilt camera motion to estimate a new pan/tilt position from a control point, assuming the camera’s intrinsic parameters and previous position are known. Chen et al. [28] combine this method with a random forest-based regression learning approach to accurately estimate the pan/tilt position in the context of a soccer field, using known 3D positions of line intersections on the playing field. Interestingly, Junejo and Foroosh [29] have shown that by taking into account the conic structure of the paths on the image plane (see Section 4.2), it is possible to improve both accuracy and noise resilience for panning or tilting cameras.

In this paper, we will further exploit the conic structure in a fundamental way. We will show how these paths can be described for a panning and/or tilting camera, even without having any prior knowledge about the location of points in the scene, the camera parameters or the initial camera pan/tilt position. By first determining the possible paths in the beginning of the sequence, the focal length and initial tilt angle of a PTZ camera can be found. This decreases the number of degrees of freedom to 1 for purely panning and 2 for simultaneous pan and tilt, as opposed to the 8 degrees of freedom in the DLT method. We will demonstrate that the background model compensation framework in [3] becomes much more robust when using the proposed techniques.

## 3. Background and Notations

**Figure 1:**
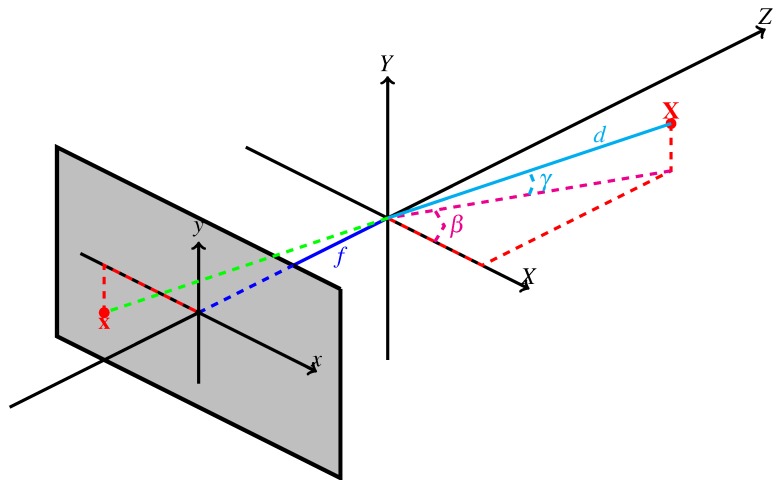
The pinhole camera model. A point X is projected on the image plane in x. *d* is the distance from X to the camera center. β and γ are the azimuthal and polar angles of the ray from X through the optical center, respectively. *f* is the focal length of the camera.

In accordance with the notations used in [24], a vector (e.g., representing the coordinates of a point) will always be represented by a bold-faced symbol. Upper case letters will be used for world coordinates, while lower case letters are used for image coordinates. The projective action of a camera is described by the following equation:

(1) wx=PX,

where *w* is a constant, $X={\left(X,Y,Z,1\right)}^{T}$ is a point in 3D space in homogeneous coordinates, $x={\left(x,y,1\right)}^{T}$ is the projection of X on the image plane in homogeneous image coordinates and *P* is the homogeneous 3 by 4 camera projection matrix. Please note that X can also be described in terms of spherical world coordinates:

(2) $${\left(X,Y,Z,1\right)}^{T}={\left(d\cos\gamma \sin\beta ,d\sin\gamma ,d\cos\gamma \cos\beta ,1\right)}^{T}\;,$$

where *d* is the Euclidean distance from the camera center to X, β is the azimuthal angle and γ is the polar angle (see Figure 1). Spherical coordinates with respect to the camera center will simplify the mathematical expressions for a rotating (panning) camera in the next section.

For a finite projective camera, *P* can be decomposed as follows [24]:

(3) $$P=KR_{0}\left[I|-C\right],\;\mathrm{where}\;K=\left[\begin{array}{ccc} f_{x} & s & x_{p}\\ 0 & f_{y} & y_{p}\\ 0 & 0 & 1 \end{array}\right]\;,$$

$R_{0}$ is a 3d rotation matrix and C is the location of the camera center, expressed in non-homogeneous world coordinates. For many real camera applications, a few reasonable assumptions [30] and coordinate choices can be made to simplify (3):

1. The skew *s* is zero.
2. The pixels are square, i.e., $f_{x}=f_{y}=f$ (the focal length).
3. The principle point is ${\left(x_{p},y_{p}\right)}^{T}={\left(0,0\right)}^{T}$ in non-homogeneous image coordinates.
4. The camera center coincides with the origin of the chosen World Coordinate System, so $C={\left(0,0,0\right)}^{T}=0_{3}^{T}$.
5. The principal axis coincides with the *Z*-axis, so the rotation matrix $R_{0}=I_{3}$, where $I_{3}$ is the (3 by 3) identity matrix.

With the above assumptions, (3) simplifies to

(4) $$P=\mathrm{diag}\left(f,f,1\right)\left[I_{3}|0_{3}^{T}\right]\;.$$

After this simplification, the mapping of a 3D point onto an image point can be written as

(5) $${\left(X,Y,Z\right)}^{T}\mapsto {\left(\frac{-fX}{Z},\frac{-fY}{Z}\right)}^{T}\;.$$

The previously described structure is well known as the ‘pinhole camera model’ (see Figure 1). This model will form the basis for the mathematical description of a panning camera in the next section.

## 4. Mathematical Description of Pan and Tilt

### 4.1. General Pan and Tilt

Most PTZ cameras are constructed such that they have two distinct rotating axes. The first rotating axis provides the panning motion, while the second one provides tilting. It is important to note here that the tilting axis is affected by panning. Hence, when the camera is simultaneously panning and tilting, the model can generally not be described by a rotation about a fixed rotational axis. However, when the tilt angle remains constant, the rotational axis remains aligned with the panning axis. This special case will be treated separately in Section 4.2. In this section, we describe the 2 degree of freedom (leaving pan and tilt angle) function that maps image points at different pan and tilt angles onto each other.

The position of the camera center can be assumed to remain static for a PTZ camera [25]. Hence, the effect of a general camera rotation on an image point x can be modeled as [24]

(6) $$x^{\prime }=Hx=KRK^{-1}x\;,$$

where $x^{\prime }$ is the transformed position of x in homogeneous coordinates, *K* is the intrinsic calibration matrix which equals diag(f,f,1) when using the pinhole camera model and *R* is a general rotation matrix in 3 dimensions. This matrix can be decomposed into several rotations along the *X*, *Y*, and *Z* axes.

The camera orientation can be defined by the current panning angle θ and the tilt angle α. Let x(θ,α) denote the projection of the same 3D point X after panning by θ and tilting by α. Let $x_{0}=x\left(0,0\right)$ denote the projection of that 3D point when the camera is at its reference position, with θ=0 and α=0. The effect of a general camera rotation on the projection of *X* is thus given by

(7) $$\begin{array}{c} x\left(\theta ,\alpha \right)=KR_{\alpha }R_{\theta }K^{-1}x_{0}\;,\\ \mathrm{where}\;\left\{\begin{array}{c} R_{\alpha }=\left[\begin{array}{ccc} \cos\alpha & 0 & -\sin\alpha \\ 0 & 1 & 0\\ \sin\alpha & 0 & \cos\alpha \end{array}\right]\\ R_{\theta }=\left[\begin{array}{ccc} 1 & 0 & 0\\ 0 & \cos\theta & -\sin\theta \\ 0 & \sin\theta & \cos\theta \end{array}\right]\;. \end{array}\right. \end{array}$$

The inverse transform is

(8) $$\begin{array}{cc} x_{0}= & KR_{\theta }^{-1}R_{\alpha }^{-1}K^{-1}x\left(\theta ,\alpha \right) \end{array}$$

(9) $$\begin{array}{cc} = & KR_{-\theta }R_{-\alpha }K^{-1}x\left(\theta ,\alpha \right)\;. \end{array}$$

Hence, a general PTZ camera motion from (θ,α) to $\left(\theta^{\prime },\alpha^{\prime }\right)$ can be decomposed as

(10) $$\begin{array}{cc} x(\theta^{\prime },\alpha^{\prime })= & KR_{\alpha^{\prime }}R_{\theta^{\prime }}K^{-1}KR_{-\theta }R_{-\alpha }K^{-1}x\left(\theta ,\alpha \right) \end{array}$$

(11) $$\begin{array}{cc} = & KR_{\alpha^{\prime }}R_{\left(\theta^{\prime }-\theta \right)}R_{-\alpha }K^{-1}x\left(\theta ,\alpha \right) \end{array}$$

(12) $$\begin{array}{cc} = & KR_{\left(\alpha +\Delta \alpha \right)}R_{\Delta \theta }R_{-\alpha }K^{-1}x\left(\theta ,\alpha \right)\;, \end{array}$$

where $\Delta \alpha =\alpha^{\prime }-\alpha$ and $\Delta \theta =\theta^{\prime }-\theta$.

In Section 5 and Section 6, methods to derive both the fixed-camera parameters and runtime camera positions will be proposed. The remainder of this section will further elaborate on the important special case where the tilt angle is fixed.

### 4.2. Panning with Fixed Tilt Angle

We first consider a single track of one specific scene point X. When the camera is purely panning with a fixed tilt angle, the apparent motion of X can be modeled by a rotation about an axis in the YZ-plane (Figure 1). If the coordinate system is assumed to rotate along with the camera, tilting the camera is equivalent to rotating points in space in the opposite direction until the rotational axis becomes aligned with the *Y*-axis:

(13) $$\left\{\begin{array}{c} X=X^{\prime }\\ Y=Y^{\prime }\cos\alpha +Z^{\prime }\sin\alpha \\ Z=-Y^{\prime }\sin\alpha +Z^{\prime }\cos\alpha \;, \end{array}\right.$$

where the new world coordinates are defined by $X^{\prime }$, $Y^{\prime }$ and $Z^{\prime }$, and α is the fixed tilt angle. Since the point X rotates in a plane with fixed $Y^{\prime }=d\sin\gamma$, the apparent motion of X can be described w.r.t these axes as

(14) $$\left\{\begin{array}{c} X^{\prime }=d\cos\gamma \sin\theta \\ Y^{\prime }=d\sin\gamma \\ Z^{\prime }=d\cos\gamma \cos\theta \;, \end{array}\right.$$

where *d* and γ remain fixed (for this scene point) and θ is the parametric rotation angle of the camera. Please note that (14) is immediately clear if we use spherical coordinates for X (see Section 3). Combining (5), (13) and (14), the apparent motion of the projection of X on the image plane can be described parametrically as

(15) $$\left\{\begin{array}{c} x=\frac{-f\cos\gamma \sin\theta }{-\sin\alpha \sin\gamma +\cos\alpha \cos\gamma \cos\theta }\\ y=\frac{-f\cos\alpha \sin\gamma -f\sin\alpha \cos\gamma \cos\theta }{-\sin\alpha \sin\gamma +\cos\alpha \cos\gamma \cos\theta }\;. \end{array}\right.$$

From this expression, we can derive the implicit equation describing the full trajectory of a point (see Appendix A):

(16) $$\begin{array}{c} A_{c}x^{2}+B_{c}y^{2}+C_{c}y+D_{c}=0\;,\\ \mathrm{where}\;\left\{\begin{array}{c} A_{c}=1-\cos\left(2\gamma \right)\\ B_{c}=-\cos\left(2\alpha \right)-\cos\left(2\gamma \right)\\ C_{c}=-2f\sin\left(2\alpha \right)\\ D_{c}=f^{2}\left(\cos\left(2\alpha \right)-\cos\left(2\gamma \right)\right)\;. \end{array}\right. \end{array}$$

This is the equation of a conic in the image plane, which represents the apparent motion of a feature point in the image when the panning camera rotates. Please note that this observation agrees with the well known property that the apparent trajectories of points on the image plane, caused by a rotating camera about an arbitrary axis through the camera center, are described by conics [24,29]. This specific form of the equation allows us to derive some important geometric properties of the trajectories:

1. The apparent motion is always symmetric regarding the *y*-axis.
2. If $A_{c}$ is strictly positive, the conic is a hyperbola whenever $B_{c}<0$, an ellipse when $B_{c}>0$ and a parabola when $B_{c}=0$, or more specifically when $\gamma +\alpha =\frac{\pi }{2}$.
3. If $A_{c}=0$ (specifically γ=0), the conic degenerates to a coincident double line.
4. Non-zero values for $C_{c}$ indicate a vertical shift.

One can observe that *d* does not appear in (15) and (16). The apparent motion is thus independent of depth. Furthermore, all rays that have the same polar angle γ (with respect to the *X*’*Y*’*Z*’ coordinate system) are projected to points that lie on a common trajectory.

**Figure 2:**
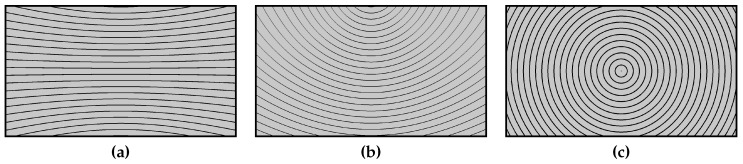
Examples of the theoretical conic shaped tracks, for different tilt angles α. (**a**) α=0, (**b**) $\alpha \;=\;\frac{\pi }{4}$, (**c**) $\alpha =\frac{\pi }{2}$.

The remaining parameters in (16) are the focal length *f* and the tilt angle α. They define the panning camera model and are thus the same for all scene points for a given camera setup. Please note that γ also does not change when the camera is panning, but it does depend on the location of the scene point being tracked. Only points in front of the camera are considered such that γ lies in $\left[-\frac{\pi }{2}-\alpha ,\frac{\pi }{2}-\alpha \right]$. Figure 2 shows notable examples for different values of the tilt angle α. In Section 5, we will demonstrate how an estimation of these fixed parameters can be obtained from a set of observed trajectories.

## 5. Panning Camera Fixed Parameter Estimation

We will first describe a scenario where the camera is purely panning and the focal length *f* and tilt angle α are still unknown. In this scenario, these parameters are both fixed. Once they are determined, *f* and α can be used in the composition of the homography for the remainder of the sequence, as will be demonstrated in Section 6. In our proposed method, the parameters are derived from analysis of feature tracks in the beginning of the sequence. Features coinciding with static background points should resemble the structure described by (16), where deviations can be explained by either unmodeled phenomena (e.g., rotational axis not through optical center, camera vibrations, lens distortion, ...) or measurement noise.

Our algorithm for finding *f* and α consists of two major steps. The first step is to determine feature points in every frame, and then track them until no more matches are found. In this work, we chose SURF features [31], since they provide robust points, while still being relatively computationally inexpensive. Please note that we expect only very small rotational and scale differences between successive frames, so in practice, also non-rotation or non-scale invariant features can be used.

**Figure 3:**
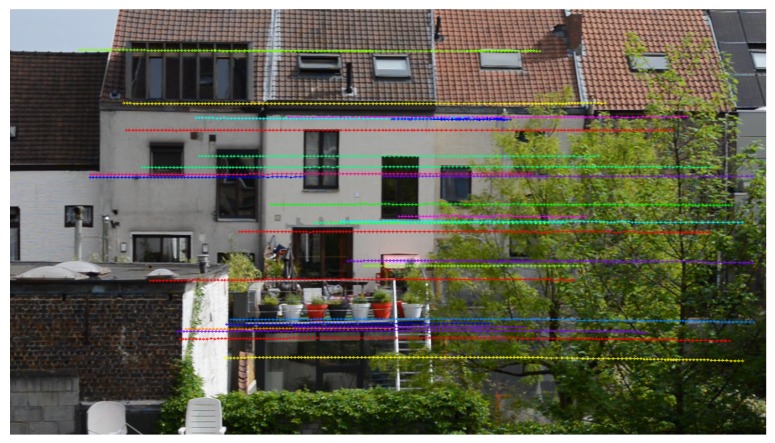
Example of feature-point tracking using SURF [31] in a panning video. The tracks of distinct feature points are visualized in different colors. Please note that tracks represent the apparent trajectory in the image, and not their motion throughout the scene.

Tracking is done by FLANN matching of points in successive frames [32], ending a track when no match is found. Furthermore, we also estimate the (unconstrained) homography with the DLT algorithm [24]. Feature correspondences that deviate too far (i.e., more than 1 pixel width) from the position calculated by the DLT are discarded. When such a large deviation occurs or when no match is found, the track is ended and stored in memory. Tracks that are too short (i.e., consisting of less than 10 points or spanning less than 10% of the entire image width) are deemed unreliable and omitted from further evaluation. An example of feature tracking in a panning camera can be found in Figure 3.

The second step consists of a nonlinear optimization to obtain good estimates for *f* and α, where the objective function to be minimized is the sum of squared distances between the observed points and the conics described by (16). Hence, when α and *f* are known, (16) determines a family of curves with parameter γ. This family represents all possible feature tracks for a panning camera with a fixed tilt angle. All points which are part of the same track *j* will possess the same polar angle $\gamma_{j}$.

Calculating the exact distance from a point to a conic is quite complex in nature [24]. In [33], an alternative cost function was proposed, which is much simpler to determine, while still providing a close approximation to the true geometric error.

The conic in (16) can be written alternatively as

(17) $$x^{T}Cx=0,\;\mathrm{where}\;C=\left[\begin{array}{ccc} A_{c} & 0 & 0\\ 0 & B_{c} & C_{c}\;/\;2\\ 0 & C_{c}\;/\;2 & D_{c} \end{array}\right]\;.$$

The *Sampson approximation* to the exact squared geometric distance between a point x and a conic, described by *C*, is defined as

(18) $${\tilde{g}}^{2}=\frac{{\left(x^{T}Cx\right)}^{2}}{4\left({\left(Cx\right)}_{1}^{2}\right)+{\left(Cx\right)}_{2}^{2})}\;,$$

where ${\left(Cx\right)}_{i}$ denotes the *i*-th component of the vector Cx. The cost function that we use consists of the sum of these squared distances (defined by (18) ) from all measured points to the conics, regarding *f*, α and γ, where γ = $\gamma_{j}$ for the *j*-th stored track. Let $x_{j}^{i}$ for $i=1\ldots M_{j}$ be $M_{j}$ points on the j-th trajectory, where *M* distinct point tracks were created in the first step. Our goal is to find α,f and $\gamma_{j}$ for 1≤j≤M such that is minimal.

(19) $$\sum_{j=1\cdots M}\sum_{i=1\cdots M_{j}}{\tilde{g}}^{2}\left(x_{j}^{i},\alpha ,f,\gamma_{j}\right))$$

Theoretically, if there are *M* distinct tracks, it is possible to simultaneously optimize *f*, α and every $\gamma_{j}$ for 1≤j≤M. However, nonlinear optimization requires finding a good initial estimate for all parameters, which is not a straightforward process in this optimization problem. Please note that this is also the reason we use (16) instead of (15) in our optimization framework, since the parameter θ (and, thus, the need to optimize its value for every feature point) is eliminated.

Luckily, finding the best estimate of the $\gamma_{j}$ values can be considered to be a distinct problem for every track, once a good estimation for *f* and α was found. Finding the best fit for a certain track *j* then only requires optimizing one parameter $\gamma_{j}$. Furthermore, from (16) follows (see Appendix B)

(20) $$\cos\left(2\gamma_{j}\right)=\sin\left(2\alpha +\phi_{j}\right)\;,\;\mathrm{where}\;\phi_{j}=\arctan\frac{f^{2}-y_{j}^{2}}{-2y_{j}f}$$

when x=0. Thus, given the y-intercept $y_{j}$ of a track and an estimate for *f* and α, we immediately obtain an estimate for $\gamma_{j}$ and the fitting cost for this particular track can be determined. All observations mentioned above are united in Algorithm 1, which we use to determine optimal values for *f* and α.

**Algorithm 1:** Determination of *f* and α for a panning camera.

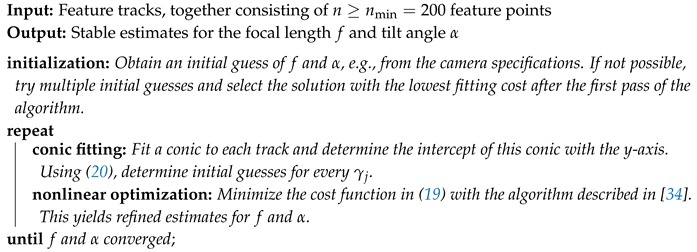



In our experiments, the algorithm always converged after only two passes, i.e., the optimization algorithm [34] detected an optimum coinciding with the initial values and, thus, returned the same values for *f* and α when more iterations were executed. The full two-pass algorithm had a total execution time of 0.6s on our hardware setup (desktop computer, single CPU).

The selection for $n_{\min}=200$ will be treated in more detail in Section 6.3, but we already briefly mention the basic idea to arrive at its value: on the one hand, when many feature points are used, a more robust estimate of the parameters can be found. On the other hand, since the parameters are not known in the beginning of the sequence, the proposed camera compensation mechanism (see Section 6 and beyond) cannot be used until *f* and α are known, which potentially sacrifices the benefits of the proposed method early on if the optimization process can only start after a large portion of the sequence has already been processed. Furthermore, in the next section we will demonstrate an update mechanism for *f* and α, to be used after their values are (initially) determined, enabling fast correction of a relatively poor initial estimate.

## 6. Pan/Tilt Camera Position Estimation

### 6.1. Panning with Fixed Tilt Angle (PFT)

Once they are known, the estimated values for *f* and α are used to determine the panning angle, i.e., the amount of azimuthal camera rotation between successive frames (Δθ). If the camera only rotates about the predefined axis, the γ of any given point also remains constant, and only the θ parameter varies. Furthermore, the calculated difference of θ between two successive frames should be the same for all points in the image. These characteristics can be exploited to calculate the panning angle between two frames, as described further in this section.

To obtain an estimate for Δθ, we observe that from (15) (see also Appendix A) the values of the azimuthal angle θ can directly be determined for any given (x,y) position:

(21) $$\tan\theta =\frac{x}{y\sin\alpha -f\cos\alpha }\;.$$

Thus, for any given pair of feature-point matches, both their panning angles θ can be calculated in that fashion, which immediately entails an estimate for the angular difference between them. We can repeat this process for any number of (robust) feature matches, and select the median of all angular differences as a robust estimate for Δθ. Hence, Algorithm 2 is used to determine the angle the camera has panned, between the times the last 2 frames were captured.

**Algorithm 2:** Determination of the panning angle between successive frames with fixed tilt.

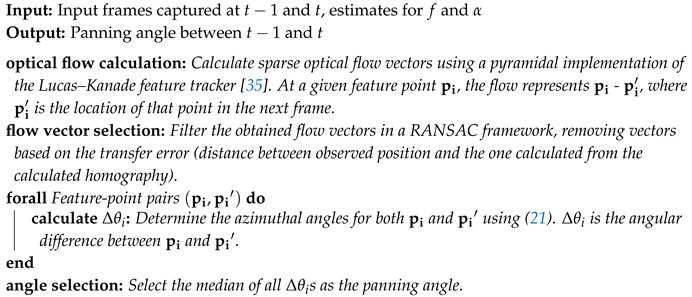



The estimated panning angle is reliable as long as the camera parameters (*f* and α) remain unchanged. In the following subsection, a model which can handle intermediate camera tilting (change in α) is explained.

### 6.2. Simultaneous Pan/Tilt (SPT)

If the tilt angle changes, the previously described rotation angle determination mechanism no longer works, since a fixed tilt angle was assumed there. In this subsection, a method which can cope with these changes is proposed, although an additional degree of freedom (related to the changing tilt angle) needs to be introduced. Note that we still assume an estimate for the initial tilt angle is known. This estimate can either be calculated with Algorithm 1 (only if the tilt angle remains fixed during this initialization phase) or requested from the camera software when available.

The goal is to obtain robust estimates for Δθ and Δα. (7) describes the position of a projected point x in terms of of α, θ, *f* and its projected position $x_{0}$ for pan and tilt angle equal to 0. A change in camera pan/tilt results in different values for θ and α, while *f* is constant. The Jacobian matrix J(θ,α), derived from this 2D function (see Appendix C for the full derivation) indicates how the position of a certain point is transformed for small Δθ and Δα:

(22) $$\begin{array}{cc} J(\theta ,\alpha )= & \left[\begin{array}{cc} dx\;/\;d\theta & dx\;/\;d\alpha \\ dy\;/\;d\theta & dy\;/\;d\alpha \end{array}\right]\\ = & \left[\begin{array}{cc} -(f+\frac{x^{2}}{f})\cos\alpha -y\sin\alpha & \frac{xy}{f}\\ -\frac{xy}{f}\cos\alpha +x\sin\alpha & f+\frac{y^{2}}{f} \end{array}\right]\;. \end{array}$$

The top and bottom row represent the change in *x* and *y* respectively. Through linearization, approximated values for the optical flow (Δx,Δy) can be found by combining the effects of Δθ and Δγ as follows:

(23) $$\begin{array}{c} \left\{\begin{array}{cc} \Delta x & \approx \frac{dx}{d\alpha }\Delta \alpha +\frac{dx}{d\theta }\Delta \theta \\ & \approx \frac{xy}{f}\Delta \alpha -\left(\left(f+\frac{x^{2}}{f}\right)\cos\alpha +y\sin\alpha \right)\Delta \theta \\ \Delta y & \approx \frac{dy}{d\alpha }\Delta \alpha +\frac{dy}{d\theta }\Delta \theta \\ & \approx \left(f+\frac{y^{2}}{f}\right)\Delta \alpha -\left(\frac{xy}{f}\cos\alpha -x\sin\alpha \right)\Delta \theta \;. \end{array}\right. \end{array}$$

Since the optical flow vectors can be calculated by a separate algorithm (e.g., [35]) and can thus be considered to be known, only two unknown variables remain in (23), which can be calculated as

(24) $$\left\{\begin{array}{cc} \Delta \alpha \approx & \frac{\left(\left(f^{2}+y^{2}\right)\Delta y-xy\Delta x\right)\cos\alpha +f\left(x\Delta x+y\Delta y\right)\sin\alpha }{\left(f^{2}+x^{2}+y^{2}\right)\left(f\cos\alpha +y\sin\alpha \right)}\\ \Delta \theta \approx & \frac{xy\Delta y-(f^{2}+y^{2})\Delta x}{\left(f^{2}+x^{2}+y^{2}\right)\left(f\cos\alpha +y\sin\alpha \right)}\;. \end{array}\right.$$

Thus, at every pixel x, Δθ and Δα can be determined in a similar manner to the purely panning camera, using Algorithm 3.

**Algorithm 3:** Determination of the panning and tilting angles between successive frames with simultaneous pan/tilt.

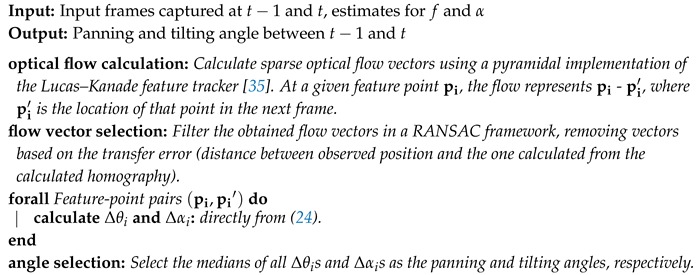



### 6.3. Increasing Robustness by Selective Parameter Updates

Up to this point, we assumed the focal length *f* and tilt angle α were perfectly estimated, and that *f* always remains unchanged throughout each sequence. For ‘fixed tilt’ sequences, we naturally also assumed α is always fixed. However, for robust practical usage it is useful to update these parameters further in the sequence. There are two main reasons to perform such an update at runtime, each requiring a different update approach:

1. These parameters can unexpectedly change (slightly) further in the sequence. The fixed tilt angle could e.g., be altered due to occurring vibrations. The focal length could for example change slightly due to refocusing on the PTZ camera itself. These parameters are now again (temporarily) ‘fixed’, but at different values from before. Since these alterations potentially occur at any given point in the sequence, it makes sense to use constant update ratios.
2. The estimated parameters generally become more accurate as more feature points are used in the optimization framework (see Section 5). However, this also leads to a slower estimation of the fixed parameters, while yielding only marginal accuracy gains at runtime from a certain point on. When the parameters are estimated more roughly and refined later, they can already provide small robustness gains earlier in the sequence. These refinements can be most effectively executed shortly after the parameters are initially estimated.

To avoid a full iterative re-estimation of *f* and α as is done in Algorithm 1, we propose an update mechanism for the fixed parameters with a much lower computational load. The general idea is to check whether better estimates for *f* and α can be found close to their current values, while the update mode can change in each frame. The objective function is the symmetric transfer error, i.e., the combined distance between the observed feature locations in the current frame and the calculated feature locations, estimated from transforming the previously observed feature points with the homography (obtained as described in Section 6.1 and Section 6.2) and vice versa.

Let *t* be the current frame and $t_{0}$ the frame on which the initial parameter estimation was done. The update mechanism is governed by

(25) $$\left\{\begin{array}{c} f_{t}=f_{t-1}+e_{f}\left(D_{f,c}+a_{f}r_{f}^{\left(t-t_{0}\right)}\right)\\ \alpha_{t}=\alpha_{t-1}+e_{\alpha }\left(D_{\alpha ,c}+a_{\alpha }r_{\alpha }^{\left(t-t_{0}\right)}\right)\;, \end{array}\right.$$

where $D_{f,c}$, $D_{\alpha ,c}$, $a_{f}$, $a_{\alpha }$, $r_{f}$ and $r_{\alpha }$ are constants. $e_{f}$ and $e_{\alpha }$ are selected from {−1,0,1}. This mechanism yields either a negative, no or a positive step with sizes $D_{f,c}+a_{f}r_{f}^{\left(t-t_{0}\right)}$ and $D_{\alpha ,c}+a_{\alpha }r_{\alpha }^{\left(t-t_{0}\right)}$ for $f_{t}$ and $\alpha_{t}$ respectively, such that the objective function is minimal. Hence, only 9 calculations of the objective function, and no computationally expensive function gradient or step size calculations are required.

One major benefit of this updating framework is its well-defined behavior. The following observations can be made:

1. If the current estimates are already sufficiently close to the optimum, $e_{f}=e_{\alpha }=0$ and the estimates for *f* and α will not be altered. Otherwise, by choosing values of ±1 for $e_{f}$ and/or $e_{\alpha }$, the values for *f* and α that provide the lowest symmetric transfer error are selected.
2. The constant terms $D_{f,c}$ and $D_{\alpha ,c}$ are primarily used to compensate changes to the ’fixed’ parameters themselves, typically taking place after the initialization period ($t\gg t_{0}$). The updating mechanism (25) then simplifies to
  (26) $$\left\{\begin{array}{c} f_{t}=f_{t-1}+e_{f}D_{f,c}\\ \alpha_{t}=\alpha_{t-1}+e_{\alpha }D_{\alpha ,c}\;. \end{array}\right.$$
  The magnitudes of $D_{f,c}$ and $D_{\alpha ,c}$ should thus be close to the magnitude of ’typical’ changes that occur to these parameters, or slightly lower for a more refined (but potentially slower) update. These deviations can of course differ for various camera setups. In our experiments, $D_{f,c}=1$ and $D_{\alpha ,c}=0.04^{{}^{\circ}}$.
3. The other terms are modeled as a geometric progression, where $r_{f},r_{\alpha }\in ]0,1[$. The first terms in these rows should be of the same order of magnitude as a typical necessary step in the optimization process. Furthermore, the sums of all elements in the row (equal to $a_{f}\;/\;\left(1-r_{f}\right)$ and $a_{\alpha }\;/\;\left(1-r_{\alpha }\right)$)should be sufficiently high to compensate the maximum remaining parameter error after the initial estimation phase. These parameters can again be optimized for different scenarios. We set $a_{f}=50$, $a_{\alpha }=2.00^{{}^{\circ}}$ and $r_{f}=r_{\alpha }=0.95$.

We shall demonstrate in Section 8 that the proposed method is notably more robust than the DLT homography technique for a relatively low number of available feature correspondences. Hence, as noted in Section 5, the earlier this benefit can be exploited, the better the overall performance of the FGBG segmentation algorithm will be. However, if the initial fixed parameters must be estimated from a limited number of (spread out) feature tracks in the optimization phase, the quality of the estimated parameters in Algorithm 1 will suffer. In that scenario, the compensation framework will provide poor results, even with the updating/refinement mechanism. Hence, a trade-off needs to be made between initial accuracy and early completion of the initialization phase. As mentioned earlier (see Algorithm 1), we found that the fixed parameter estimation framework could optimally be initiated with tracks consisting of a total number of $n_{\min}=200$ feature points in our experiments. Depending on the dataset, selecting a different number can yield slightly better overall results.

## 7. Integration into FGBG Segmentation

**Figure 4:**
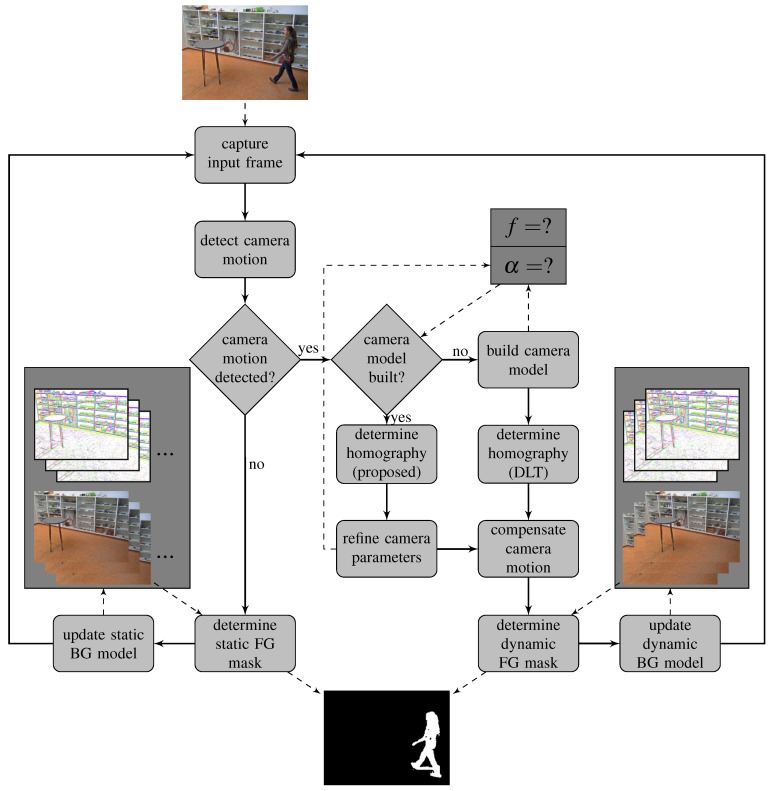
Flowchart demonstrating the internal operation of the foreground–background segmentation algorithm. The camera model is presented in Section 3 and Section 4. Building the camera model is described in detail in Section 5. The estimation of the homography and camera parameter refinement are treated in Section 6. The other blocks are handled as described in [3] and [24]. Transitions from one block to another are denoted in solid lines, whereas dashed lines refer to internal or external input/output operations.

The proposed techniques can be used to extend many (classical) FGBG segmentation methods that assume the camera position remains static, by transforming the background model according to the transformation formulas obtained in Section 5 and Section 6. Since C-EFIC [3] currently achieves the highest $F_{1}$ score of any unsupervised method on the full PTZ category for the ChangeDetection.NET 2014 dataset [22], but still uses a different compensation mechanism (based on affine transformation), we will demonstrate our approach by incorporating the proposed methods to that model. This also allows for a fair evaluation of the performance of the PTZ compensation method itself, as opposed to other potential improvements such the use of alternative image features. An overview of the full foreground–background segmentation method is shown in Figure 4.

C-EFIC uses two distinct background models, both combining RGB color and LTP (Local Ternary Pattern [36]) feature samples, stored at every pixel location individually. Pixel locations where not enough (<2) background samples are close (up to a distance threshold) to the current input in feature space are considered to be foreground, as was first proposed in [13].

The first model assumes a static camera, and is built over a relatively long period. 25 feature samples of both RGB and LTP features are stored locally. The learning rate decreases over time, while dynamic regions (e.g., swinging tree branches) are also detected automatically and updated faster than static ones (e.g., empty street). This model has the benefit that complex distributions of background appearances can be stored, but it cannot cope with dynamic camera viewpoints.

The second, dynamic model is only used when camera motion is detected. The dynamic model consists of fewer samples (3 instead of 25 in our experiments), and is transformed after every frame. Please note that in C-EFIC, the foreground-detection step explained above also checks the background model for all pixels in the 8-neighborhood surrounding that pixel. This method can cope with small transformation errors, but slowly removes (static) true foreground objects from the mask as a downside. This mechanism was not necessary in our proposed method thanks to the superior compensation mechanism, whereas it did prove beneficial for C-EFIC.

In the original C-EFIC method, a reliable transformation is determined using RANSAC to estimate an affine transformation matrix. One benefit of this method is that no prior information concerning the camera is required. However, this transformation is not able to accurately transform the model when the camera is panning or tilting, as was explained in Section 2. In our proposed method, we replace the affine compensation with the DLT homography compensation during the initialization phase. As soon as a camera motion is detected, tracks of SURF features are stored as described in Section 5. The available tracks are then analyzed and the fixed parameters are estimated. After this, in every frame, the panning angle (Section 6.1) or both the panning and tilting angle (Section 6.2) of the camera can be estimated, depending on which is selected by the user. To ensure a robust estimate, the initialization phase only stops when enough feature tracks are found, consisting of a total of at least $n_{\min}$ points.

The background model needs to be transformed after every frame to compensate for the pan and tilt motion between the last two frames. Areas which were previously outside of the field of view are automatically added to the background model. Lanczos resampling is used for storing the new background samples, since this method preserves the image content better than classical interpolation methods such as nearest neighbor or bilinear interpolation [37]. Once the panning motion stops, the samples in the dynamic model are copied into the static model, and the learning rate is reset.

The full algorithm is coded in C++ and runs at approximately 3 fps for 640 by 480-pixel video sequences on a desktop computer, using a single CPU. We will demonstrate that both proposed compensation mechanisms (used in their respective applications) are superior to the affine compensation in our experiments. Compared to an unconstrained homography, our proposed method is also able to produce better results when a low number of feature matches is used, as we will demonstrate in the following section.

## 8. Experiments

The pan/tilt compensation mechanisms are evaluated against unconstrained affine and homography estimation techniques. Next, the proposed algorithm is embedded into the foreground–background segmentation framework described in [3], and the algorithm is tested on 10 different scenarios. An overview of the parameters used in all experiments is given in Table 1.

### 8.1. Pan/tilt Camera Position Estimation

For these experiments, we first recorded 6 videos, looking onto a static scene. Hence, all image points can be considered background. All tests were performed on real images, recorded from a mounted Axis P5624-E camera. The camera was tilted downward with angles of 0°, 5°, 10°, 15°, 20° and 25°. These values were set using the camera software. The zoom factor was kept constant in all sequences, although autofocusing was enabled. On these sequences, our proposed methods for both panning with fixed tilt and simultaneous pan/tilt were evaluated. *f* and α are first estimated online by using the techniques described in Section 5.

Furthermore, we recorded 4 additional sequences: 1 where the panning angle remains constant and only tilt changes occur and 3 where a human operator was given the instruction to pan and tilt randomly. In these last 3 videos, the operator was also given the instruction to use either slow, medium, or fast camera movements, respectively. Since the tilt angle is no longer fixed, we omit the method described in Section 6.1 from the analysis here.

Theoretically, all evaluated methods, except the affine compensation, should be able to fully compensate for a purely panning camera or a simultaneously panning and tilting camera, respectively. In practice, the observed feature-point matches (Section 6) diverge from the models they use, e.g., due to image noise or motion blur. Errors made by the compensation mechanisms can be partially mitigated by considering more feature points to estimate the transformation. However, this leads to slower processing, but more importantly it cannot be guaranteed that enough stable discriminative features can be found in the scene (e.g., in scenes where large regions contain little texture). Thus, compensation methods become robust if they can produce reliable results when the number of calculated feature matches is low.

**Figure 5:**
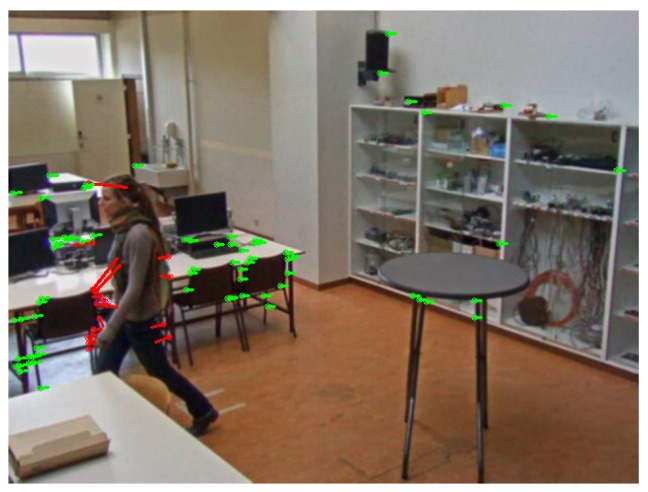
Example of matches found through sparse optical flow matching [35], where the features are selected through the feature detector described in [38]. Points coinciding with foreground locations in the previous frame are discarded immediately. RANSAC is then used to further eliminate outliers (red). The remaining matches (green) are employed to determine the panning/tilting motion of the camera.

**Figure 6:**
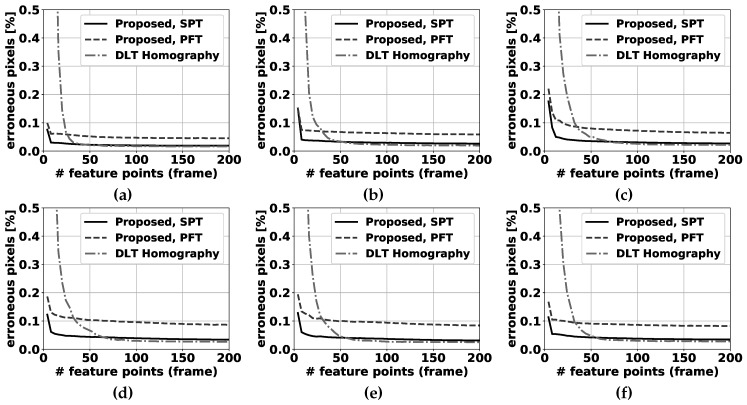
Percentage of erroneous pixels in fixed tilt sequences where feature points are selected by using the algorithm described in [38]. The proposed panning for fixed tilt (PFT) method and the simultaneous pan and tilt (SPT) method are compared to the DLT homography method [24] for 6 sequences: (**a**) 0° tilt, (**b**) 5° tilt, (**c**) 10° tilt, (**d**) 15° tilt, (**e**) 20° tilt, (**f**) 25° tilt. Please note that the number of erroneous pixels is significantly above 0.5% of the total number of pixels for a low number of feature correspondences, and cannot be clearly depicted on these graphs.

**Figure 7:**
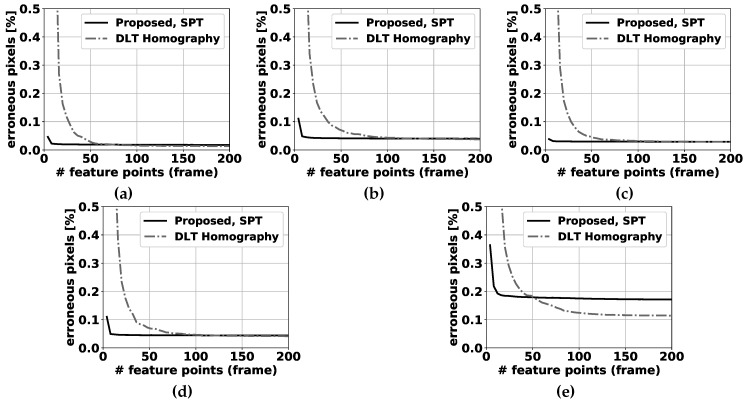
Percentage of erroneous pixels in variable tilt sequences where feature points are selected by using the algorithm described in [38]. The proposed panning for simultaneous pan and tilt (SPT) method is compared to the DLT homography method [24] for 5 sequences: (**a**) pure tilt, (**b**) equal pan and tilt, (**c**) random pan/tilt (slow), (**d**) random pan/tilt (medium), (**e**) random pan/tilt (fast). Please note that the number of erroneous pixels is significantly above 0.5% of the total number of pixels for a low number of feature correspondences, and cannot be clearly depicted on these graphs.

For selecting feature points in the compensation step, the feature detector described in [38] is used. The transformation between all successive input frame pairs (I(t),I(t−1)) is estimated using our proposed method, the unconstrained homography calculated by the direct linear transformation (DLT, described in [24]) with nonlinear optimization refinement and the affine transformation as used in [3], each resulting in a separate transformation matrix. Then, I(t−1) is transformed by the obtained matrices, and compared with I(t) pixelwise. If the difference is larger than a fixed threshold, this pixel is marked as an erroneous transformed pixel. The best camera motion compensation method obviously has the lowest number of badly transformed points. Please note that the threshold is chosen equal to the RGB difference threshold in the FGBG segmentation method described in [3]. In that fashion, the number of erroneous transformed pixels is directly related to the number of false positive pixels expected to occur in the eventual foreground images. Furthermore, to ensure a realistic and fair comparison between the different methods, RANSAC (based on the estimated pan + tilt homography, cfr. Algorithm 3) was first applied to filter all obtained feature correspondences, and the exact same remaining pairs were used as input for each method (see Figure 5). Results for the fixed tilt angle sequences can be found in Figure 6, while Figure 7 demonstrates results for the variable tilt sequences.

In general, the more feature points are available, the lower the number of erroneous pixels for all methods will be. However, the marginal gains decrease exponentially as more feature points are used. For the fixed tilt sequences, the proposed method for simultaneous pan and tilt (SPT) demonstrates slightly better performance than the fixed tilt method (PFT). This leads us to the conclusion that the addition of a degree of freedom for the change in tilt angle is slightly beneficial regarding the PFT method, even if the camera software was only given the instruction to pan the camera. This can be attributed to vibrations that occur in real camera setups, and to potential small errors in the distortion removal process: when camera distortion is not fully or correctly removed, feature tracks will also be distorted and one additional d.o.f. can partially overcome compensation errors accompanying this scenario. The unconstrained DLT homography method [24] performs notably worse than both proposed methods when few feature matches are available. In 5 sequences, this is clearly visible in the results for less than 50 matches and below 30 matches in the remaining 0° tilt sequence for the SPT method, and below 25 matches for the PFT method. When more feature points are used, the performance differences between the proposed SPT method and the DLT homography are negligible, with a slight advantage for the DLT homography.

For the variable tilt sequences, we compared our SPT method with the DLT homography. Similar conclusions can be drawn as for the fixed tilt sequences, with one notable exception. When the camera pan/tilt speed is relatively high ($0.8^{{}^{\circ}}$ of rotation on average per frame, see Figure 7e), the performance of our proposed method is notably worse than the DLT homography’s above 50 feature matches. This can be attributed to the usage of linearization (see Section 6.2): the bigger the distance between the matched point pairs, the worse these expressions approximate the true feature tracks. For a lower number of feature points, the proposed method still clearly outperforms the DLT homography.

**Figure 8:**
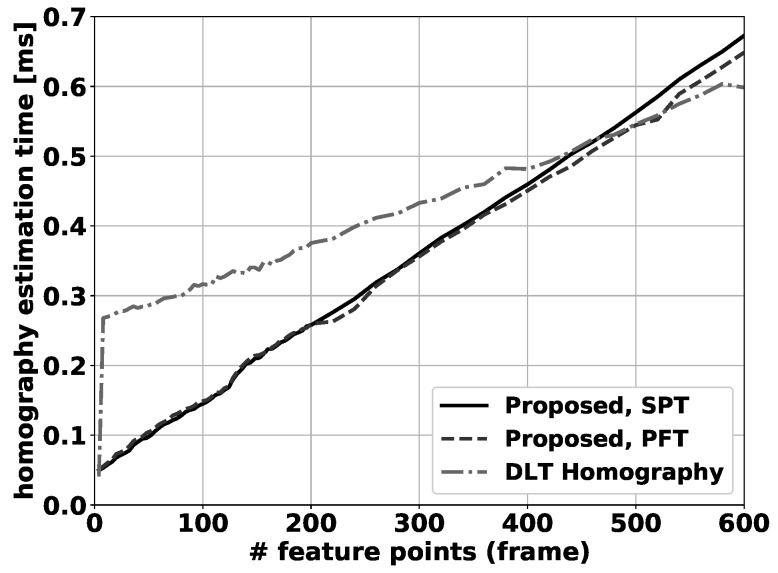
Average homography calculation time per frame, as a function of the number of feature points used in the calculation.

A processing speed comparison between the different compensation methods is shown in Figure 8. When only 4 feature points are used in the calculation, all methods have similar processing times. Above that, the DLT homography method (OpenCV implementation) must perform additional calculations to estimate the homography in a nonlinear framework, which explains the big jump in processing time beyond 4 feature points, where both proposed methods are notably faster. For more than 500 calculated feature matches, the homography method becomes faster than the proposed method, although in practical applications far fewer feature matches are typically available (or used) as noted before. The differences between both proposed methods are small, with the fixed tilt method being marginally faster, most notably for a high number of feature-point matches. One can observe that the overall processing time for the compensation step is still very low (3 orders of magnitude smaller for 250 feature points) compared to the overall foreground background segmentation algorithm, which runs at approximately 3 frames per second on the same hardware setup.

### 8.2. Foreground–Background Segmentation

The full foreground–background segmentation method was tested on 9 different self-captured videos and one publicly available sequence, which will be treated separately. The authors plan to make the self-captured dataset available on request.

**Figure 9:**
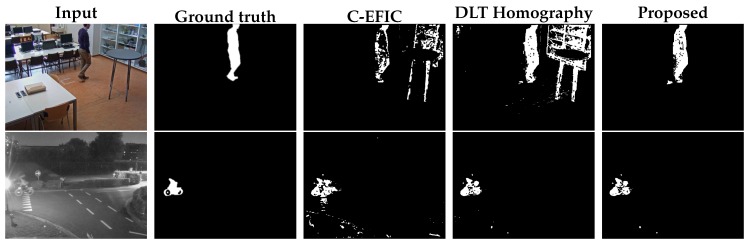
Foreground–background segmentation result for two handpicked fixed tilt panning frames, using 12 feature points per frame. Top row: indoor sequence, bottom row: outdoor night sequence input.

**Figure 10:**
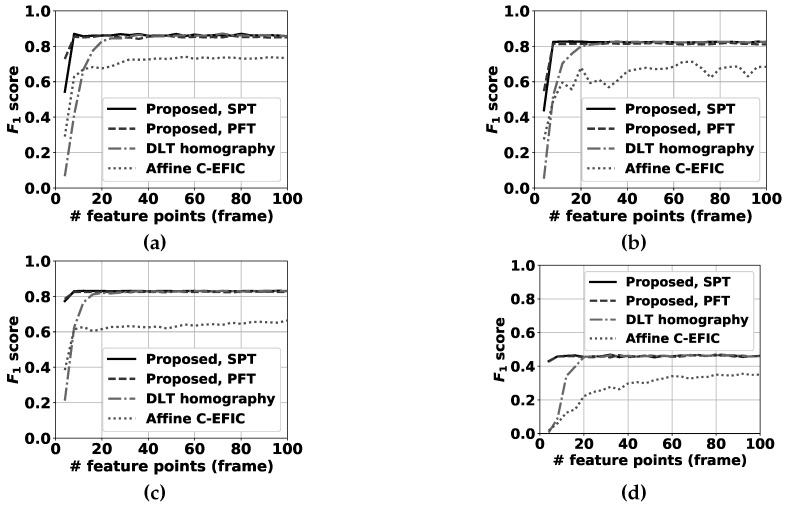
$F_{1}$ scores for the full FGBG segmentation method in the fixed tilt sequences. The proposed methods for panning with fixed tilt (PFT) method and the simultaneous pan and tilt (SPT) method are compared to the DLT homography method [24] and the affine compensation mechanism used before in C-EFIC [3] for 4 sequences; (**a**) classroom 5° tilt, (**b**) classroom 15° tilt, (**c**) classroom 25° tilt, (**d**) parking outdoor night.

Out of the self-captured videos, the tilt angle was kept fixed in 4 sequences. These sequences consist of 3 indoor videos with people walking around in a computer class environment and 1 outdoor video. For the indoor sequences, the camera was tilted to 5°, 15° and 25°. The outdoor sequence is notably challenging, as it was captured at a parking lot at night, containing reflections and generally difficult illumination conditions. Camera distortion was calculated with the algorithm described in [39] and removed as a preprocessing step. Visual examples can be found in Figure 9. For every sequence, one frame was manually annotated as ground truth for every second of video. In total, 229 frames were annotated and successively analyzed. Results for the proposed fixed tilt compensation method as well as for the DLT and original affine compensation [3] can be found in Figure 10.

The proposed methods are again notably more robust for a low number of feature matches, as can be expected from the results in Section 8.1. However, the differences between the individual methods are much less pronounced in the $F_{1}$ score. Also, for 4 feature matches, the PFT method is superior to the SPT method, but above that the SPT method yields slightly better results. Once above 20 feature-point matches, the compensation error differences between the individual homography methods become negligible compared to the inherent $F_{1}$ score fluctuations introduced by the random background update mechanism. However, compared to the original C-EFIC method, the proposed methods are clearly superior for any given number of feature matches. Please note that the difficult night sequence yields lower $F_{1}$ scores overall compared to the other sequences.

**Figure 11:**
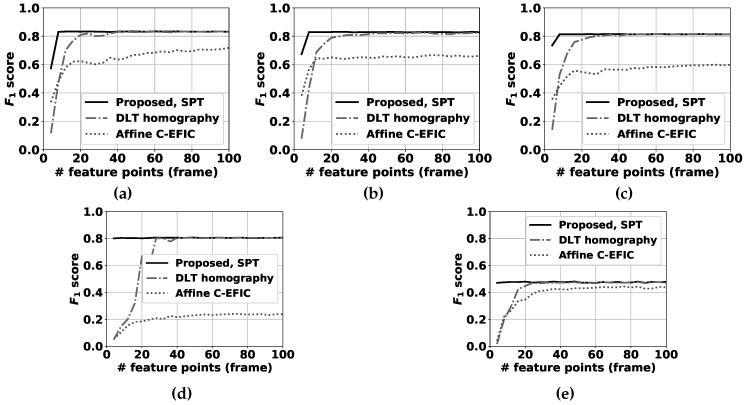
$F_{1}$ scores for the full FGBG segmentation method in the variable tilt sequences. The proposed method for simultaneous pan and tilt (SPT) is compared to the DLT homography method [24] and the affine compensation mechanism used before in C-EFIC [3] for 5 sequences; (**a**) classroom pan/tilt 1, (**b**) classroom pan/tilt 2, (**c**) classroom pan/tilt 3, (**d**) street outdoor day (**e**) parking outdoor night.

**Figure 12:**
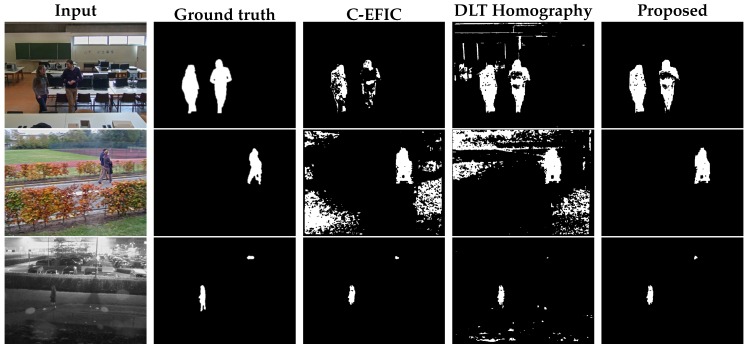
Foreground–background segmentation result for three handpicked simultaneous pan/tilt frames, using 12 feature points per frame. Top row: indoor sequence, middle row: outdoor day sequence, bottom row: outdoor night sequence.

The self-captured simultaneous pan/tilt sequences again captured different scenarios where people are walking around in a classroom environment indoor (3 videos). Furthermore, 2 outdoor sequences were recorded, where the camera monitored a street at daytime and a parking lot at night, respectively. 430 frames were annotated and analyzed in total. Numerical results of the foreground–background segmentation can be found in Figure 11. Visual results are shown in Figure 12.

Similar to the scenario where the camera is only panning, the proposed pan/tilt method and the DLT-based method show superior performance compared to the original C-EFIC affine method in the processed sequences. Also, the proposed method is again superior to the DLT method for a low number of feature points, while comparable results can be obtained when the number of feature matches becomes larger.

**Figure 13:**
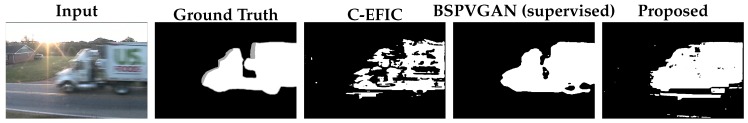
Foreground–background segmentation results for a handpicked frame from the publicly available ‘continuousPan’ sequence. Links to other methods can be found on the ChangeDetection.NET website ([22]).

Finally, the proposed method was evaluated on the most relevant sequence in the public ChangeDetection.NET 2014 dataset, i.e., the fixed tilt ‘continuousPan’ sequence. Unlike in the self-captured videos, camera distortion was not removed, because the dataset only provides the undistorted test video and no distortion parameters. A fixed number of 50 feature points was selected in every frame. The proposed PFT method is compared to relevant state-of-the-art methods (both supervised and unsupervised) for whom the resulting foreground masks were available. We note that when using 50 feature points, using the DLT Homography or the proposed SPT method should yield very similar performance metrics. Quantitative results can be found in Table 2. A visual example is demonstrated in Figure 13.

At the moment of writing, the proposed foreground–background segmentation method currently produces the best $F_{1}$ score of any unsupervised method currently available for this sequence. Furthermore, the performance for this sequence is only slightly inferior to the one submitted for two state-of-the-art supervised methods. Only FgSegNet_S [40] still performs significantly better, though it should be noted that this method was trained from a manual selection of ground truth images contained in the dataset itself.

### 8.3. Discussion

The previous sections demonstrate that the proposed methods are superior to the unconstrained DLT homography for a low number of feature points, both in processing speed and compensation reliability. For a higher number of feature points (e.g., in a textured region), the DLT homography (with additional nonlinear optimization) yields comparable compensation results, or slightly superior ones when the camera rotation speed is high. Furthermore, the proposed SPT method (see Section 6.2) is slightly superior the PFT method even for fixed tilt sequences, although this is not fully noticeable in the $F_{1}$ scores for the full foreground background segmentation method.

This demonstrates that imposing constraints and reducing the number of degrees of freedom from 8 to 2 (representing pan + tilt) can definitely increase the robustness of a camera motion compensation framework. The second degree of freedom (related to tilt) should probably not be discarded for a panning camera and for most practical applications, the SPT method should be preferred.

In comparison to other FGBG segmentation methods, the proposed method demonstrates state-of-the-art unsupervised performance on a publicly available test sequence, and still achieves near state-of-the-art results when compared to the tested supervised segmentation methods. This experiment also demonstrates that even without offline camera distortion removal, the proposed pan/tilt camera motion model can still be robust. In such an event, the (initial) camera parameter estimation step in our proposed algorithm estimates a ‘distorted’ focal length and initial tilt angle, such that it matches the image content better. For this sequence, the deviation from the theoretical model due to distortion was largely mitigated in that manner.

It should be noted that is still possible to improve segmentation results for background subtraction-based methods in general by also taking semantic information into account, as was demonstrated by Braham et al. [41]. This should yield a more accurate segmentation of typical foreground objects, such as pedestrians, bicycles, and cars. Finally, the segmentation masks resulting from unsupervised methods can still serve as initializations to high-performing semi-supervised methods, as was noted by Wehrwein and Szeliski [8]. The foreground mask of the proposed method could replace the (otherwise necessary) manually annotated masks, potentially leading to a similar performance if the selected foreground masks are accurate.

## 9. Conclusions and Future Work

In this paper, we demonstrated a method to compensate a panning and tilting camera motion, used in the application of foreground–background segmentation. A mathematical framework of the camera motion was constructed, using the pinhole camera model. Next, feature tracks obtained in the beginning of a sequence are used to determine the necessary intrinsic parameters. From that point in time, the model can be used to accurately estimate the camera motion occurring between two frames.

A first compensation method assuming panning with fixed tilt (PFT) was shown to robustly compensate for the motion of a panning camera, especially compared to affine motion compensation. For a small number of used feature matches, this proposed algorithm is also able to compensate the background model notably better than the full 8 degree of freedom DLT homography method.

Furthermore, a framework for a simultaneously panning and tilting (SPT) camera was proposed, which yields even slightly better results with respect to the fixed panning method. When a high number of feature points is used, the SPT method still yields a performance similar to the DLT homography method. In the context of foreground–background segmentation, these tendencies are visible in the obtained $F_{1}$ scores. When the proposed framework is used to replace the affine compensation mechanism, that algorithm’s performance is notably increased for any given number of feature matches in our experiments. The full foreground–background segmentation method achieves state-of-the-art unsupervised results on the ‘continuousPan’ sequence of the publicly available ChangeDetection.NET 2014 dataset. When supervised methods are taken into account, the $F_{1}$ scores are still close to those that state-of-the-art methods are capable of.

In principle, the method can be extended to incorporate the zooming function, which translates to varying values for *f* in the camera model. Since any combination of such a rotation and zooming can still be compensated by a homography, it is possible to determine the parameters in a similar manner to the one described in this paper. However, the compensation mechanism will likely be more complex, since additional parameters need to be introduced.

Situations where the camera center is moved throughout the scene (e.g., in cameras mounted on vehicles) need a different approach. Though similar equations of feature tracks for particular motions can be constructed, the homography can no longer be used and effects such as parallax need to be taken into account.

## Figures and Tables

**Table 1:** Overview of the values of all parameters mentioned in this manuscript and used in our experiments. For the parameters related to the C-EFIC method, we refer to [3].

Parameter	Value
$n_{\min}$	200
$D_{f,c}$	1
$a_{f}$	50
$r_{f}$	0.95
$D_{\alpha ,c}$	0.04°
$a_{\alpha }$	2.00°
$r_{\alpha }$	0.95

**Table 2:** Comparison of Precision, Recall and $F_{1}$ scores obtained by the proposed method and state-of-the-art methods on the ’continuousPan’ sequence of the public ChangeDetection.NET 2014 dataset. These scores were obtained with the software provided by the creators of the dataset. Supervised methods are denoted with an ‘S’. Links to other methods can be found on the ChangeDetection.NET website ([22]).

Method	Precision	Recall	$F_{1}$ Score
Proposed	0.8827	0.8894	0.8860
FgSegNet_S (S)	0.9688	0.9889	0.9787
BSPVGAN (S)	0.8673	0.9776	0.9191
cascade CNN (S)	0.8421	0.9666	0.9001
DeepBS (S)	0.1037	0.9103	0.1862
C-EFIC	0.7133	0.6432	0.6764
MBS	0.7907	0.5257	0.6315
CwisarDRP	0.2952	0.6082	0.3975
PAWCS	0.1953	0.7316	0.3083
IUTIS-5	0.1814	0.4922	0.2651

## Abbreviations

The following abbreviations are used in this manuscript:

PTZ	Pan-Tilt-Zoom
FGBG	Foreground–Background
MoG	Mixture of Gaussians
DLT	Direct Linear Transformation
PFT	Panning with Fixed Tilt
SPT	Simultaneous Pan/Tilt

## Appendix A. Derivation of Panning Camera Equation

Starting from the parametric Equation (15), the canonical equation of the apparent trajectory of a point can be derived as follows:

(A1) $$\left\{\begin{array}{c} x=\frac{-f\cos\gamma \sin\theta }{-\sin\alpha \sin\gamma +\cos\alpha \cos\gamma \cos\theta }\\ y=\frac{-f\cos\alpha \sin\gamma -f\sin\alpha \cos\gamma \cos\theta }{-\sin\alpha \sin\gamma +\cos\alpha \cos\gamma \cos\theta } \end{array}\right.$$

(A2) $$\Leftrightarrow \left\{\begin{array}{c} \cos\theta =\frac{(y\sin\alpha -f\cos\alpha )\sin\gamma }{(y\cos\alpha +f\sin\alpha )\cos\gamma }\\ \sin\theta =\frac{x\sin\gamma }{(y\cos\alpha +f\sin\alpha )\cos\gamma }\;. \end{array}\right.$$

Using the trigonometric identity ($\cos^{2}\theta +\sin^{2}\theta =1$), the parameter θ can be eliminated:

(A3) $${\left[\frac{(y\sin\alpha -f\cos\alpha )\sin\gamma }{(y\cos\alpha +f\sin\alpha )\cos\gamma }\right]}^{2}+{\left[\frac{x\sin\gamma }{(y\cos\alpha +f\sin\alpha )\cos\gamma }\right]}^{2}=1\;.$$

Reworking this equation further yields:

(A4) $$x^{2}\left[\sin^{2}\gamma \right]+y^{2}\left[\sin^{2}\alpha \sin^{2}\gamma -\cos^{2}\alpha \cos^{2}\gamma \right]-y\left[2f\sin\alpha \cos\alpha \right]+f^{2}\left(\cos^{2}\alpha \sin^{2}\gamma -\sin^{2}\alpha \cos^{2}\gamma \right)=0$$

(A5) $$\Leftrightarrow x^{2}\left[1-\cos\left(2\gamma \right)\right]-y^{2}\left[\cos\left(2\alpha \right)+\cos\left(2\gamma \right)\right]-y\left[2f\sin\left(2\alpha \right)\right]+f^{2}\left(\cos\left(2\alpha \right)-\cos\left(2\gamma \right)\right)=0$$

(A6) $$\Leftrightarrow A_{c}x^{2}+B_{c}y^{2}+C_{c}y+D_{c}=0\;,\;\mathrm{where}\;\left\{\begin{array}{c} A_{c}=1-\cos\left(2\gamma \right)\\ B_{c}=-\cos\left(2\alpha \right)-\cos\left(2\gamma \right)\\ C_{c}=-2f\sin\left(2\alpha \right)\\ D_{c}=f^{2}\left(\cos\left(2\alpha \right)-\cos\left(2\gamma \right)\right)\;. \end{array}\right.$$

## Appendix B. Derivation of *γ* from *y*-Intercept, *f* and *α*

Starting from (A6), cos(2γ) can be isolated as follows:

(A7) $$\begin{array}{cc} \cos(2\gamma )= & \frac{x^{2}+\left(f^{2}-y^{2}\right)\cos\left(2\alpha \right)-2yf\sin\left(2\alpha \right)}{x^{2}+f^{2}+y^{2}} \end{array}$$

(A8) $$\begin{array}{cc} = & \frac{x^{2}+\sqrt{{\left(f^{2}-y^{2}\right)}^{2}+{\left(-2yf\right)}^{2}}\sin\left(2\alpha +\phi \right)}{x^{2}+f^{2}+y^{2}} \end{array}$$

(A9) $$\begin{array}{cc} = & \frac{x^{2}+\left(f^{2}+y^{2}\right)\sin\left(2\alpha +\phi \right)}{x^{2}+f^{2}+y^{2}}\;, \end{array}$$

where $\phi =\arctan\frac{f^{2}-y^{2}}{-2yf}$. Thus, if x=0, the previous equation simplifies to

(A10) cos(2γ)=sin(2α+ϕ).

## Appendix C. Derivation of Pan/Tilt Camera Jacobian

From (7):

(A11) $$x\left(\theta ,\alpha \right)=KR_{\alpha }R_{\theta }K^{-1}x_{0}\;,\;\mathrm{where}\;\left\{\begin{array}{c} R_{\alpha }=\left[\begin{array}{ccc} \cos\alpha & 0 & -\sin\alpha \\ 0 & 1 & 0\\ \sin\alpha & 0 & \cos\alpha \end{array}\right]\\ R_{\theta }=\left[\begin{array}{ccc} 1 & 0 & 0\\ 0 & \cos\theta & -\sin\theta \\ 0 & \sin\theta & \cos\theta \end{array}\right]\;. \end{array}\right.$$

Expressions for x and y can be derived:

(A12) $$\left\{\begin{array}{c} x=f\frac{x_{0}\cos\theta -\sin\theta }{\left(\cos\theta +x_{0}\sin\theta \right)\cos\alpha -y_{0}\sin\alpha }\\ y=f\frac{\left(\cos\theta +x_{0}\sin\theta \right)\sin\alpha +y_{0}\cos\alpha }{\left(\cos\theta +x_{0}\sin\theta \right)\cos\alpha -y_{0}\sin\alpha }\;. \end{array}\right.$$

The inverse transformation yields:

(A13) $$\left\{\begin{array}{c} x_{0}=\frac{(f\cos\alpha +y\sin\alpha )\sin\theta +x\cos\theta }{(f\cos\alpha +y\sin\alpha )\cos\theta -x\sin\theta }\\ y_{0}=\frac{y\cos\alpha -f\sin\alpha }{(f\cos\alpha +y\sin\alpha )\cos\theta -x\sin\theta }\;. \end{array}\right.$$

Hence, by taking partial derivatives of (A12) and inserting the expression for $x_{0}$ and $y_{0}$ in (A12), the Jacobian matrix *J* can be calculated:

(A14) $$\begin{array}{cc} J(\theta ,\alpha )= & \left[\begin{array}{cc} dx\;/\;d\theta & dx\;/\;d\alpha \\ dy\;/\;d\theta & dy\;/\;d\alpha \end{array}\right] \end{array}$$

(A15) $$\begin{array}{cc} = & \left[\begin{array}{cc} -(f+\frac{x^{2}}{f})\cos\alpha -y\sin\alpha & \frac{xy}{f}\\ -\frac{xy}{f}\cos\alpha +x\sin\alpha & f+\frac{y^{2}}{f} \end{array}\right]\;. \end{array}$$

## References

[1] Yi K.M., Yun K., Kim S.W., Chang H.J., Choi J.Y. Detection of moving objects with non-stationary cameras in 5.8ms: Bringing motion detection to your mobile device. Proceedings of the IEEE Conference on Computer Vision and Pattern Recognition Workshops.

[2] Kim D.S., Kwon J. (2016). Moving object detection on a vehicle mounted back-up camera. Sensors.

[3] Allebosch G., Van Hamme D., Deboeverie F., Veelaert P., Philips W. (2016). C-EFIC: Color and edge based foreground background segmentation with interior classification. Computer Vision, Imaging and Computer Graphics Theory and Applications.

[4] Deng J., Dong W., Socher R., Li L.J., Li K., Li F.F. ImageNet: A large-scale hierarchical image database. Proceedings of the IEEE Conference on Computer Vision and Pattern Recognition (CVPR).

[5] Tsai Y.H., Yang M.H., Black M.J. Video segmentation via object flow. Proceedings of the IEEE Conference on Computer Vision and Pattern Recognition (CVPR).

[6] Caelles S., Maninis K.K., Pont-Tuset J., Leal-Taixé L., Cremers D., Van Gool L. One-Shot Video Object Segmentation. Proceedings of the IEEE Conference on Computer Vision and Pattern Recognition (CVPR).

[7] Perazzi F., Khoreva A., Benenson R., Schiele B., Sorkine-Hornung A. Learning Video Object Segmentation from Static Images. Proceedings of the IEEE Conference on Computer Vision and Pattern Recognition (CVPR).

[8] Wehrwein S., Szeliski R. Video Segmentation with Background Motion Models. Proceedings of the BMVC.

[9] Stauffer C., Grimson W.E.L. Adaptive background mixture models for real-time tracking. Proceedings of the IEEE Computer Society Conference on Computer Vision and Pattern Recognition.

[10] Bouwmans T., El Baf F., Vachon B. (2008). Background Modeling Using Mixture of Gaussians for Foreground Detection—A Survey. Recent Pat. Comput. Sci..

[11] Cristani M., Farenzena M., Bloisi D., Murino V. (2010). Background subtraction for automated multisensor surveillance: A comprehensive review. EURASIP J. Adv. Signal Process..

[12] Kim K., Chalidabhongse T.H., Harwood D., Davis L. Background Modeling and Subtraction by Codebook Construction. Proceedings of the International Conference on Image Processing (ICIP).

[13] Barnich O., Van Droogenbroeck M. (2011). ViBe: A universal background subtraction algorithm for video sequences. IEEE Trans. Image Process..

[14] Van Droogenbroeck M., Paquot O. Background subtraction: Experiments and improvements for ViBe. Proceedings of the IEEE Conference on Computer Vision and Pattern Recognition (CVPR) Workshops.

[15] Grünwedel S., Petrovic N., Jovanov L., Niño Castañeda J., Pizurica A., Philips W. (2013). Efficient foreground detection for real-time surveillance applications. Electron. Lett..

[16] Heikkila M., Pietikainen M. (2006). A texture-based method for modeling the background and detecting moving objects. IEEE Trans. Pattern Anal. Mach. Intell..

[17] St-Charles P.L., Bilodeau G.A., Bergevin R. (2015). SuBSENSE: A universal change detection method with local adaptive sensitivity. IEEE Trans. Image Process..

[18] St-Charles P.L., Bilodeau G.A., Bergevin R. A self-adjusting approach to change detection based on background word consensus. Proceedings of the IEEE Winter Conference on Applications of Computer Vision (WACV).

[19] Allebosch G., Van Hamme D., Deboeverie F., Veelaert P., Philips W. Edge based foreground background estimation with interior/exterior Classification. Proceedings of the 10th International Conference on Computer Vision Theory and Applications.

[20] Hofmann M., Tiefenbacher P., Rigoll G. Background segmentation with Feedback: The Pixel-Based Adaptive Segmenter. Proceedings of the IEEE Computer Society Conference on Computer Vision and Pattern Recognition Workshops.

[21] Mittal A., Huttenlocher D. Scene modeling for wide area surveillance and image synthesis. Proceedings of the IEEE Conference on Computer Vision and Pattern Recognition (CVPR).

[22] Wang Y., Jodoin P.M., Porikli F., Konrad J., Benezeth Y., Ishwar P. CDnet 2014: An Expanded Change Detection Benchmark Dataset. Proceedings of the IEEE Conference on Computer Vision and Pattern Recognition (CVPR) Workshops.

[23] Komagal E., Yogameena B. (2018). Foreground segmentation with PTZ camera: A survey. Multimed. Tools Appl..

[24] Hartley R.I., Zisserman A. (2004). Multiple View Geometry in Computer Vision.

[25] Hayman E., Murray D.W. (2003). The Effects of Translational Misalignment when Self-Calibrating Rotating and Zooming Cameras. IEEE Trans. Pattern Anal. Mach. Intell..

[26] Wu Y.C., Chiu C.T. Motion clustering with hybrid-sample-based foreground segmentation for moving cameras. Proceedings of the IEEE International Conference on Acoustics, Speech and Signal Processing (ICASSP).

[27] Li Y., Zhang J., Hu W., Tian J. (2015). Method for pan-tilt camera calibration using single control point. J. Opt. Soc. Am. A Opt. Image Sci. Vis..

[28] Chen J., Zhu F., Little J.J. A Two-Point Method for PTZ Camera Calibration in Sports. Proceedings of the 2018 IEEE Winter Conference on Applications of Computer Vision (WACV).

[29] Junejo I.N., Foroosh H. (2012). Optimizing PTZ Camera Calibration from Two Images. Mach. Vis. Appl..

[30] de Agapito L., Hartley R.I., Hayman E. Linear self-calibration of a rotating and zooming camera. Proceedings of the IEEE Computer Society Conference on Computer Vision and Pattern Recognition.

[31] Bay H., Ess A., Tuytelaars T., Van Gool L. (2008). Speeded-Up Robust Features (SURF). Comput. Vis. Image Underst..

[32] Muja M., Lowe D.G. Fast approximate nearest neighbors with automatic algorithm configuration. Proceedings of the VISAPP International Conference on Computer Vision Theory and Applications.

[33] Sampson P.D. (1982). Fitting conic sections to “very scattered” data: An iterative refinement of the bookstein algorithm. Comput. Graph. Image Process..

[34] Transtrum M.K., Sethna J.P. (2012). Improvements to the Levenberg-Marquardt Algorithm for nonlinear least-squares minimization. arXiv.

[35] Bouguet J.Y. Pyramidal Implementation of the Lucas Kanade Feature Tracker Description of the Algorithm. https://pdfs.semanticscholar.org/aa97/2b40c0f8e20b07e02d1fd320bc7ebadfdfc7.pdf.

[36] Tan X., Triggs B. (2010). Enhanced Local Texture Feature Sets for Face Recognition Under Difficult Lighting Conditions. IEEE Trans. Image Process..

[37] Turkowski K. (1990). Graphics Gems.

[38] Shi J., Tomasi C. Good features to track. Proceedings of the IEEE Conference on Computer Vision and Pattern Recognition.

[39] Zhang Z. (2000). A Flexible New Technique for Camera Calibration. IEEE Trans. Pattern Anal. Mach. Intell..

[40] Lim L.A., Keles H.Y. (2018). Foreground segmentation using convolutional neural networks for multiscale feature encoding. Pattern Recognit. Lett..

[41] Braham M., Piérard S., Van Droogenbroeck M. Semantic background subtraction. Proceedings of the IEEE International Conference on Image Processing (ICIP).

